# Towards sensible toxicity testing for nanomaterials: proposal for the specification of test design

**DOI:** 10.1088/1468-6996/16/6/065006

**Published:** 2015-12-09

**Authors:** Annegret Potthoff, Mirco Weil, Tobias Meißner, Dana Kühnel

**Affiliations:** 1 Fraunhofer Institute for Ceramic Technologies and Systems, Winterbergstrasse 28, D-01277 Dresden, Germany; 2 ECT Oekotoxikologie GmbH, Boettgerstrasse 2-14, D-65439 Floersheim, Germany; 3 Helmholtz Centre for Environmental Research—UFZ, Dept. Bioanalytical Ecotoxicology, Permoserstrasse 15, D-04318 Leipzig, Germany; 4 Present address: Institut für Korrosionsschutz Dresden GmbH, Gostritzer Strasse 65, D-01217 Dresden, Germany.; 5 Author to whom any correspondence should be addressed.

**Keywords:** decision support, harmonization, physical-chemical characterisation, hazard assessment, engineered nanomaterials, toxicity test design

## Abstract

During the last decade, nanomaterials (NM) were extensively tested for potential harmful effects towards humans and environmental organisms. However, a sound hazard assessment was so far hampered by uncertainties and a low comparability of test results. The reason for the low comparability is a high variation in the (1) type of NM tested with regard to raw material, size and shape and (2) procedures before and during the toxicity testing. This calls for tailored, nanomaterial-specific protocols. Here, a structured approach is proposed, intended to lead to test protocols not only tailored to specific types of nanomaterials, but also to respective test system for toxicity testing. There are existing standards on single procedures involving nanomaterials, however, not all relevant procedures are covered by standards. Hence, our approach offers a detailed way of weighting several plausible alternatives for e.g. sample preparation, in order to decide on the procedure most meaningful for a specific nanomaterial and toxicity test. A framework of several decision trees (DT) and flow charts to support testing of NM is proposed as a basis for further refinement and in-depth elaboration. DT and flow charts were drafted for (1) general procedure—physicochemical characterisation, (2) choice of test media, (3) decision on test scenario and application of NM to liquid media, (4) application of NM to the gas phase, (5) application of NM to soil and sediments, (6) dose metrics, (S1) definition of a nanomaterial, and (S2) dissolution. The applicability of the proposed approach was surveyed by using experimental data retrieved from studies on nanoscale CuO. This survey demonstrated the DT and flow charts to be a convenient tool to systematically decide upon test procedures and processes, and hence pose an important step towards harmonisation of NM testing.

## 1. Introduction

During the last decade, enormous effort has been put into the assessment of potential adverse effects of engineered nanomaterials (ENMs) for both humans and the environment. However, up to now a sound hazard assessment is often hampered by results that are difficult to compare and may be contradictory [1]. This is often due to [1] missing information about the nanomaterial identity and characteristics [2], interaction of NM with the particular test media and the dispersion procedure applied [3], unpredictable behaviour of NM during the test and [4] difficulties in dose metrics and data interpretation.

So far there is no generally applicable model for identification of hazards of nanomaterials and scientists as well as regulatory committees recommend a case by case approach for hazard assessment [2, 3]. The high variability of different nanomaterials with regard to raw material, size and shape (among others) calls for tailored, nanomaterial-specific protocols.

At present, several studies suggest that the registration, evaluation, authorisation and restriction of chemicals approach is suitable for hazard assessment and risk characterisation nanomaterials [4]. Concerning the performance of toxicological tests the Organisation for Economic Co-operation and Development (OECD) test guidelines comprise internationally agreed testing methods. Tests performed according to these guidelines are useful for both risk assessment and classification purposes [5]. But the specific properties of nanomaterials are not considered in test guidelines developed for usual chemicals, and disregarding them was shown to lead to undesired interferences between nanomaterials and abiotic and biotic factors in the test e.g. [6–9]. Hence, though generally appropriate for testing of nanomaterials, methodological modifications of these guidelines may be necessary [10]. The inadequacies for testing of nanomaterials are mostly related to material characterisation, properties and metrology [11, 12]. These findings suggest that extensive modification of OECD test guidelines are unnecessary and nanomaterial-specific issues might be addressed in guidance documents on how existing test guidelines could be used in testing of nanomaterials.

Accordingly, as a first resource to reach this goal, a structured approach employing decision trees (DT) and flow charts is proposed here to guide through the process of choosing appropriate testing schemes for a specific type of NM. The suite of decisions trees and flow charts developed summarise the main considerations for setting up a well-designed toxicity test for nanomaterials. They are flexible in starting point and extent, and have been suggested by several scientists to be a valuable tool in nanotoxicology [12–14]. As the resulting decisions are material- and toxicity test dependent, the trees illustrate the hierarchical, consecutive decisions, without weighting alternative approaches. The final decision on which way to choose is left to expert knowledge depending on the intended testing goal. Recently, a DT-like approach has been used to assign the hazard potential to specific NM in relation to the intended use [15].

The applicability domain of the structured approach developed here comprises all nanomaterials irrespective of their raw materials, under the precondition that they are both toxicological relevant and dispersible to a degree acceptable for toxicological assays, for example with regard to aggregate size and wettability. Two test scenarios are considered, referred to as worst-case (optimised for stable NM dispersions) and realistic scenario (testing under relevant conditions, accepting agglomeration of NM). To minimise the main causes of variations during testing as described above, the following DT and flow charts were drafted: (1) general procedure—physicochemical characterisation, (2) choice of test media, (3) decision on test scenario and application of NM to liquid media, (4) application of NM to the gas phase, (5) application of NM to soil and sediments, (6) dose metrics, (S1) definition of a nanomaterial, and (S2) dissolution. They were developed in accordance with current regulations and definitions for NM.

The major aims of this work were to (1) provide a specific, yet variable frame for NM type specific testing, (2) provide a stepwise approach to design NM toxicity tests and in consequence (3) provide guidance and foster harmonisation in testing. Not only results from current analysis will be more reliable, reproducible and comparable, but also the retrospective analysis of existing data with regard to their reliability is conceivable.

In order to assess the applicability of the proposed approach, the procedure was tested using experimental data retrieved from studies on nanoscale CuO (nCuO). The DT were then used to define the testing schemes leading to most reliable results while considering the intended test scenario. They were shown to be a convenient tool to systematically structure test procedures and processes, and hence to be suitable to define a reliable test design. Thus, the DT and flow charts presented here provide a basal, superordinate procedure to structure the workflow and measures to be taken based on ENM specific, toxicity test specific, but also research question specific considerations. Accordingly, they may also provide a structured approach to develop standard operating procedures (SOPs) for the testing of NM. They can hence be seen as a backbone for testing and may be extended and further specified in the future.

## 2. Approach and methods

### 2.1. Drafting of DT and flow charts

A DT is a tool using a tree-like graph or model of decisions and their possible consequences to help identify a strategy most likely to reach a specified goal. A flow chart is a representation of the sequence single steps are performed in order to reach a specific goal or solve a problem.

The DT and flow charts apply to all ENMs irrespective of their raw material given that they are both toxicological relevant and dispersible to a degree acceptable for toxicological assays, for example with regard to aggregate size and wettability. DT and flow charts were drafted in an iterative process taking into account practical experience from earlier experimental procedures and existing standards [16, 17]. Furthermore, the DT were developed to be applicable for several toxicological test systems.

### 2.2. Example study involving experimental data on nanoscale CuO

**Figure 1.**
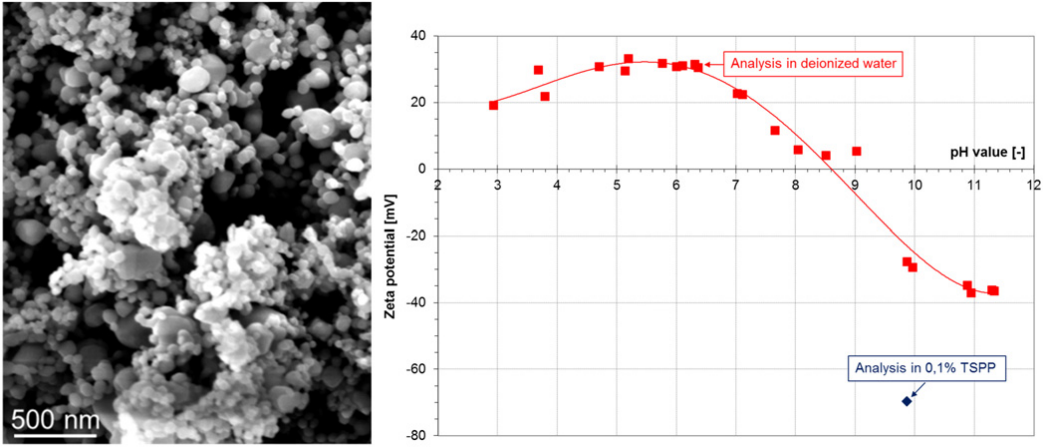
SEM image of CuO (NNV-011). Zeta potential in dependence on pH value (red squares), as well as in presence of 0.1% TSPP (blue diamond).

The structured approach was tested for applicability, suitability and comprehensiveness by choosing one type of ENM. CuO (NNV-011, delivered by Nanologica AB) powder contains nanoscaled primary particles, forming submicronscaled aggregates, which represent the smallest dispersable unit. The main NM properties and characteristics of the powder are summarised in table 1, and a scanning electron microscopy (SEM) image is shown in figure 1.

**Table 1.** Relevant parameters of the studied nCuO (BET—Brunauer–Emmett–Teller, DLS—dynamic light scattering).

Parameters	CuO NNV-011	CuO NNV-011 + 0.1% TSPP
CAS number		1317-38-0
Chemical composition, crystallinity		CuO, contains <5% Cu_2_O
Specific surface area (BET)		27 m^2^ g^−1^
primary particle size *x*_BET_		35 nm
Particle size in stock suspension after dispersion (at *t* = 0; smallest dispersable unit) *x*_DLS_	144 ± 5 nm	136 ± 5 nm
Zeta potential in stock suspension	+31 mV	−69 mV

**Figure 2.**
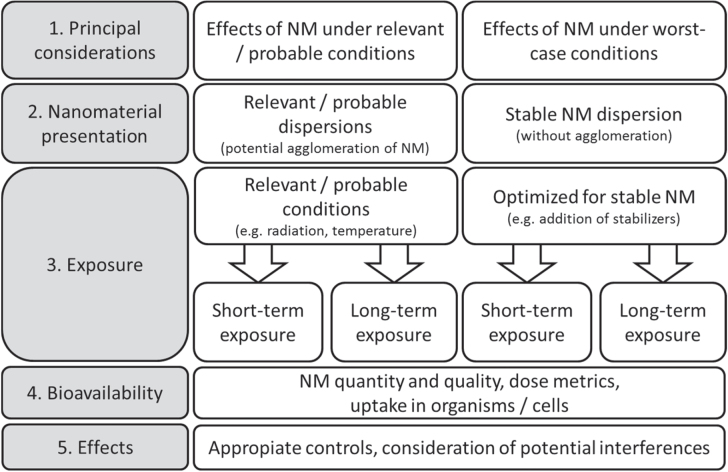
Principal consideration for the testing of nanomaterials (modified after [22]).

The purpose was to cover different initial states of NM such as powders and suspensions, and different testing scenarios (see figure 2). For example, different states of agglomeration may be used in an experiment, which defines how much effort is put into the dispersion and stabilisation of a NM. Since the properties of nanomaterials strongly depend on their surroundings the following two test scenarios were compared.

(1) *Daphnia magna* immobilisation test according to OECD guideline 202.

The test guideline is well-established for the testing of chemicals; however adaptations are required specific to NM testing. Sample preparation, in particular the dispersion of powder in liquid may determine the test results. Two different approaches will be considered: (a) use of dispersant aids to improve the colloid chemical stability of the suspension, such as polyphosphates e.g. [18–20]; (b) the same particle concentration, energy input etc as in (a) are chosen, but the test will be conducted without dispersants. A modified Daphnia medium (ADaM) was used for organisms exposure (after [21]). Approach (a) will involve stabilized nanomaterials, assuming that organisms are exposed to smaller sized particles compared to approach (b). Accordingly, approach (a) was termed ‘worst-case’-scenario, whereas approach (b) was termed ‘realistic’ scenario, as organisms will more likely contact the agglomerates.

(2) *Daphnia magna* immobilisation test conducted in real waters.

Scenario (1) represents a well-defined test involving artificial test media. To cover further testing approaches, the same test protocol was followed but exposure was conducted in real waters from two surface water sources of different composition. The waters (named water 1 and water 2) differed in relevant parameters such as dissolved matter and chloride content as well as conductivity. Especially the latter one determines the behaviour of CuO particles in media and is connected with ionic strength. ADaM shows the highest amount of soluble salts and hence, it improves the tendency for particle agglomeration.

The properties of the applied test media for these tests are summarised in table 2.

**Table 2.** Relevant characteristics of test media and natural waters used in the example studies. (DOC—dissolved organic carbon.)

	Test according to OECD guideline	Lake water I	Lake water II
DOC (mg C l^−1^)	—	3.4	1.1
pH	7.4	8.2	8.3
Ca^2*^ (mg l^−1^)	73.7	48.6	37.4
Cl^−^ (mg l^−1^)	267.2	23.7	1.6
Conductivity (*μ*S cm^−1^)	997	390	200

## 3. Results

### 3.1. DT and flow charts

**Figure 3.**
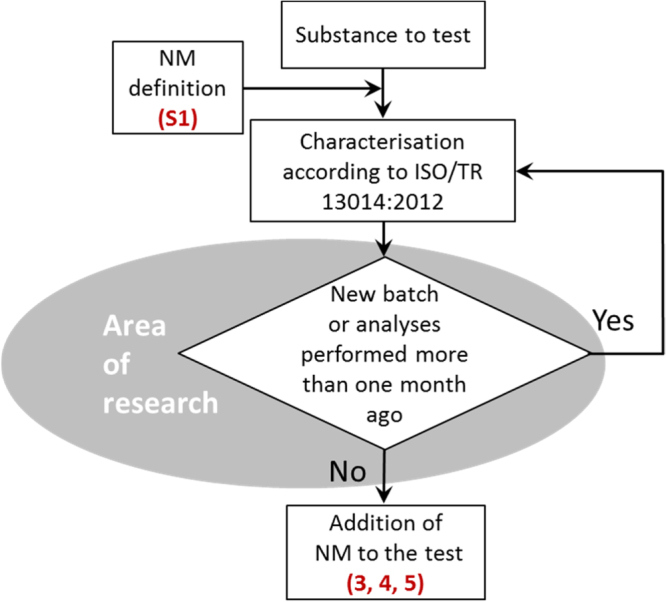
Flow chart 1: general procedures—physicochemical characterisation of the nanomaterial. Numbers given in parenthesis relate to the detailed decision trees shown in figures S1, 5–7. More specification of the phenomena underlying the aging process is needed, hence this is marked as area of research.

**Figure 4.**
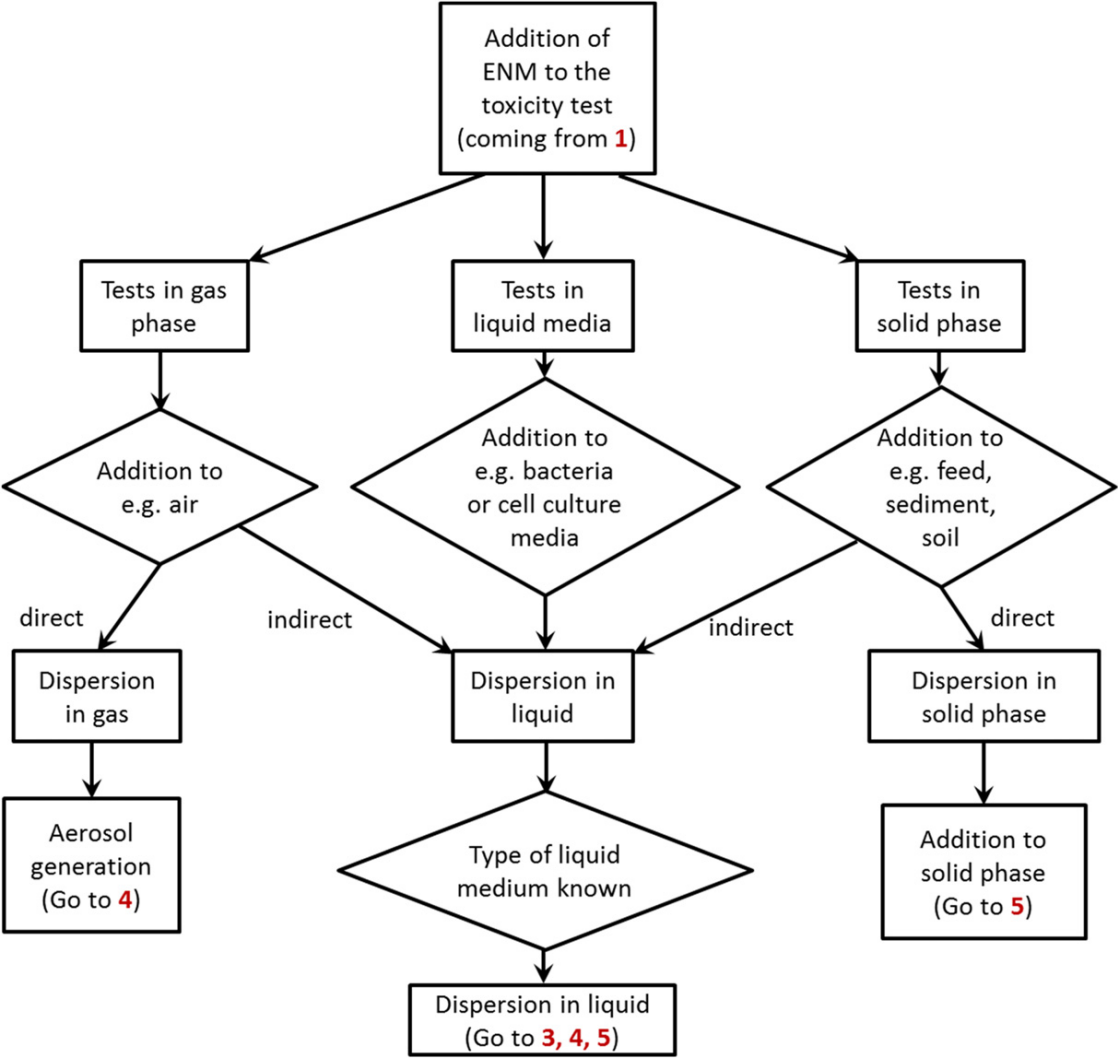
Flow chart on the choice of test media and considerations on dosing. Numbers given in parenthesis relate to the detailed decision trees shown in figures 5–7.

**Figure 5.**
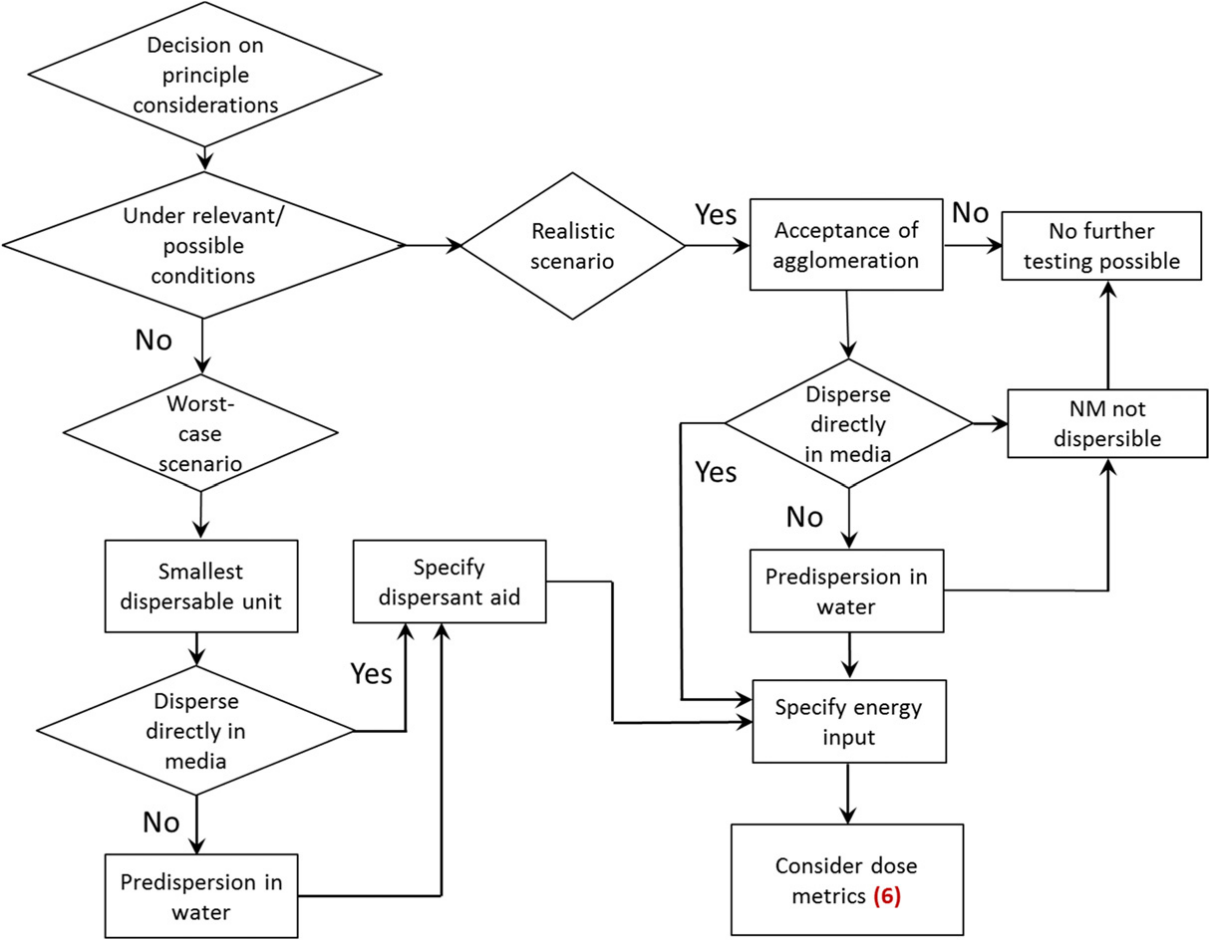
Decision tree 3: choice of test scenario and application of NM to liquid media. Number given in parenthesis relate to the decision tree shown in figure 8.

**Figure 6.**
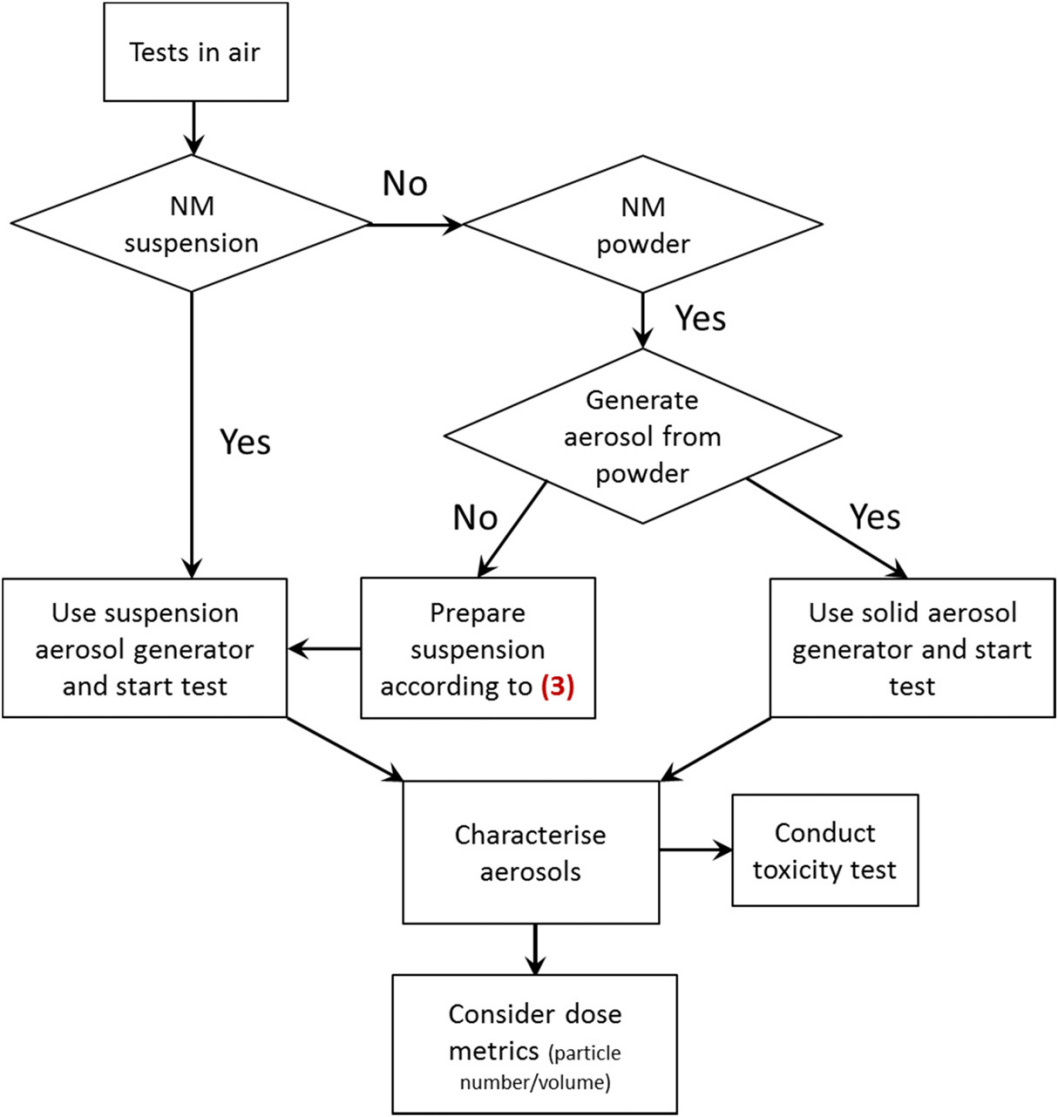
Decision tree 4: application of nanomaterials to the gas phase. Number given in parenthesis relate to the decision trees shown in figure 5.

**Figure 7.**
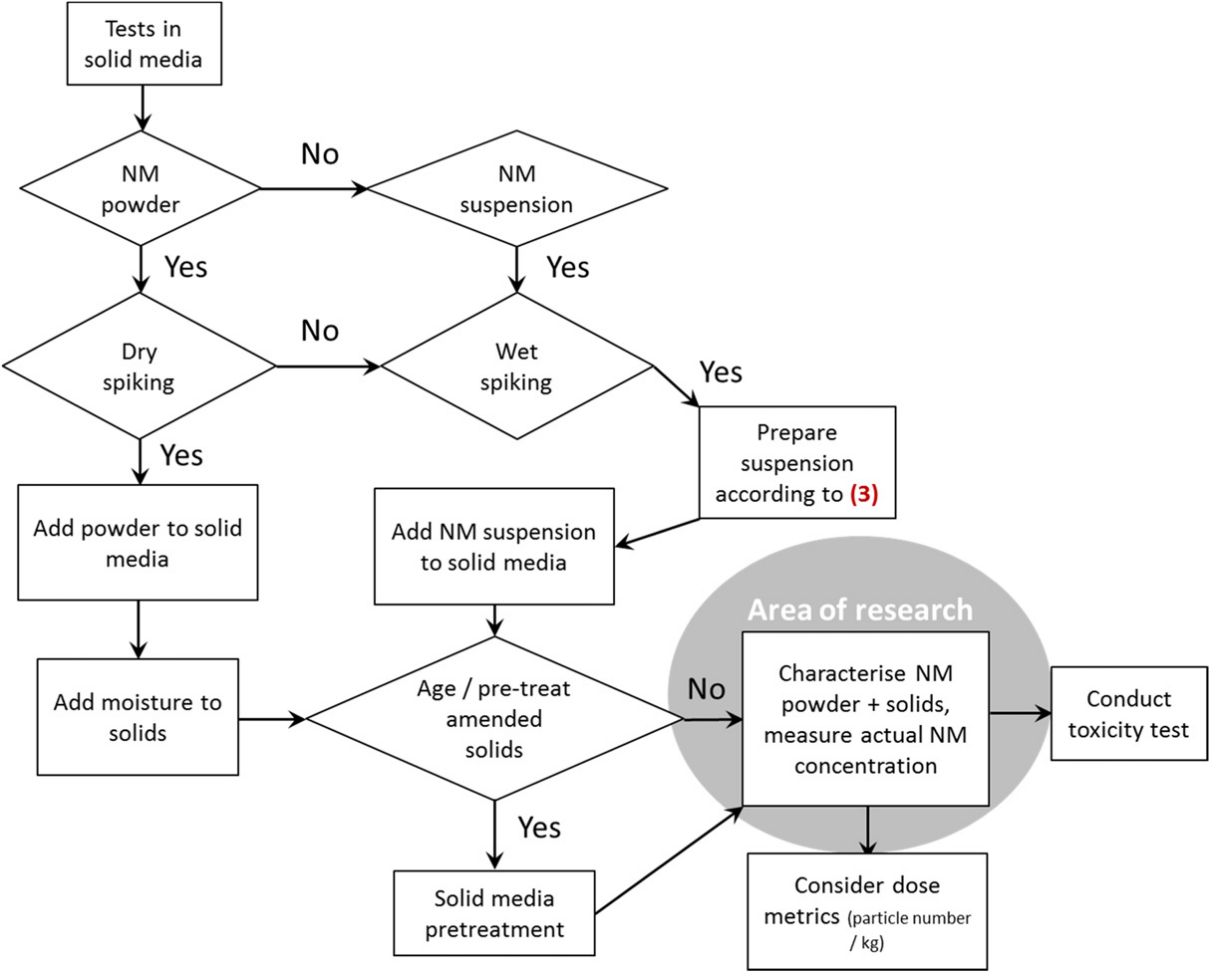
Decision tree 5: application of nanomaterials to solid test media. Number given in parenthesis relate to the decision tree shown in figure 5.

**Figure 8. F0008:**
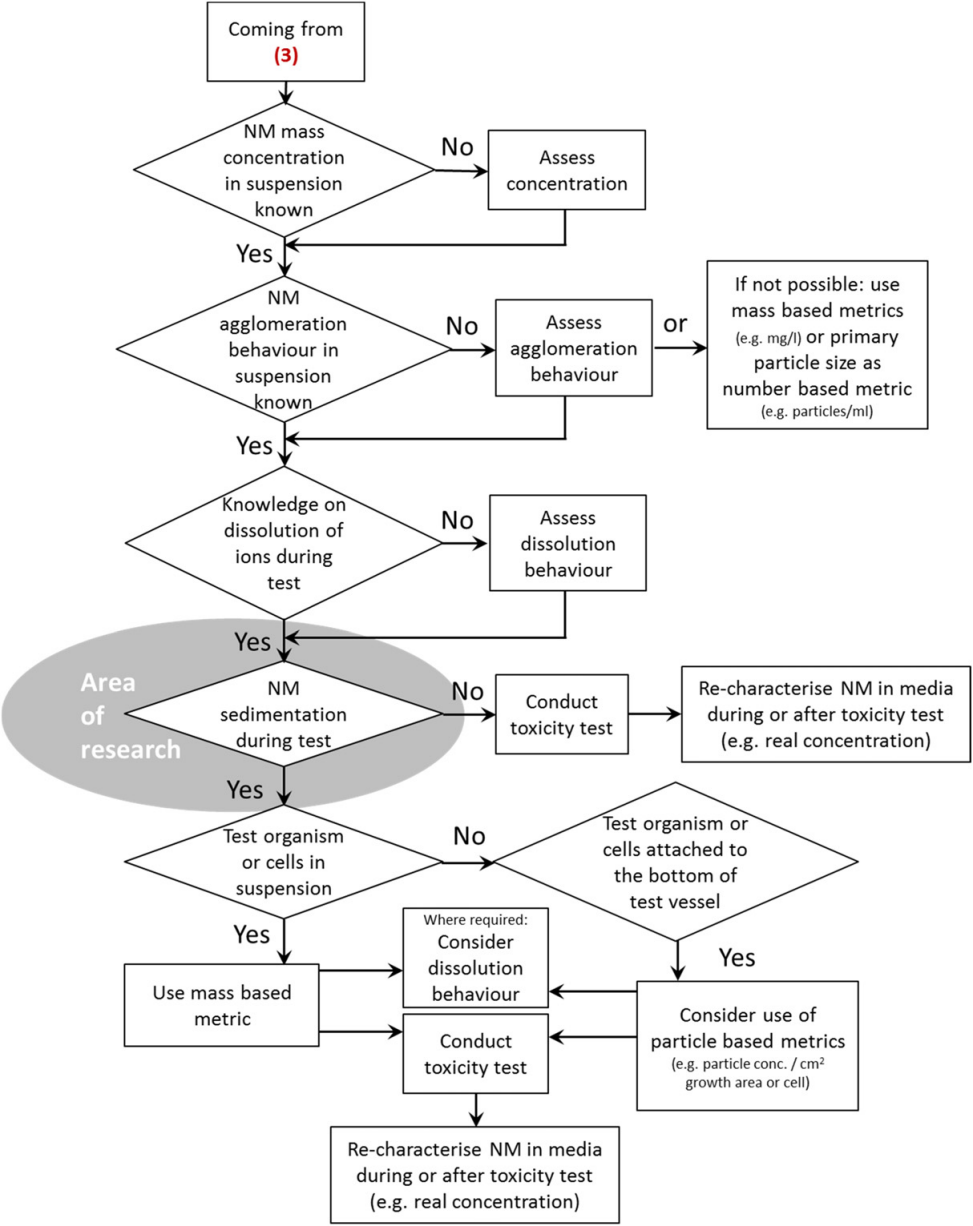
Decision tree 6: dose metrics in liquid media. The decision node on sedimentation is marked as ‘area of research’, as currently no protocols for a reliable quantification of the amount of nanomaterials settled out during the tests are available. Numbers given in parenthesis relate to the decision tree on liquid media shown in figure 5.

In total, six DT and two flow charts supporting the toxicity testing of NM were developed: (flow chart 1) general procedure—physicochemical characterisation, (flow chart 2) choice of test media, (DT 3) decision on test scenario and application of NM to liquid media, (DT 4) application of NM to the gas phase, (DT 5) application of NM to soil and sediments, (DT 6) dose metrics (see figures 3–8), (DT S1) definition of a nanomaterial, and (DT S2) dissolution (see supporting information). They were developed in concordance with current regulations and definitions for NM (ISO, 2008, ISO, 2010). The area of applicability of the DT is depicted in detail in tree S1 NM definition (supplement S1).

The general procedures are summarised in figures 3 and 4. Depending on the type of NM and the specific tests under consideration, the trees are linked with each other as indicated in the figures.

For each specific DT and flow chart, data and pre-assessments are required in order to reach the final decision. The required data can either be deduced from manufactures information, or have to be assessed by the researcher choosing among various methods in order to proceed and make relevant decisions for the test system. Some data are difficult to obtain for NM, e.g. behaviour in soil and sediment; hence, data gaps and methodological limitations are likewise identified and reported. Finally, the major outputs and the way to proceed with other trees are explained.

### 3.2. Flow chart (1) general procedure—physicochemical characterisation of the original NM

An unknown powder or a suspension is to be tested. As the following procedure mainly covers testing of nanoscaled materials, the first step (S1) is to determine whether the material is a nanomaterial. If this is verified, the testing proceeds with the physicochemical characterisation as shown in figure 3.

This flow chart describes the initial characterisation of a pristine/as-delivered nanomaterial according to ISO/TR 13014:2012. It involves the assessment of powder or suspension characteristics such as particle size.

Special attention has to be paid to changes of nanomaterials properties under storage and test conditions. In general, due to the influence of parameters such as temperature, light, pH or due to interactions with vessels for storage, nanomaterials may change their over time. Therefore, especially for nanodispersions it is necessary to repeat characterisation procedures before further (toxicological) testing, in case a longer time period has passed between these two steps. This improves the interpretation of toxicological effects from different studies and improves the comparability of test results e.g. [23].

Additional important aspects to consider are effects from variations occurring during the production process of the NM. Various batches of the same material may differ in chemical properties (as content of impurities or thickness of coating) or in physical properties (as strength of agglomerates or aggregates) [24, 25]. As a result, different batches of the same material may need to be investigated separately. Hence, the knowledge of initial NM characteristics is considered as indispensable. However, more research is needed to determine the crucial parameters and time-dependency for aging of specific nanomaterials. This is marked in figure 3 as ‘area of research’.

Dispersion is defined as the homogenous introduction of solid particles into gas, liquids or solids, by applying one or more of various mixing methods. During the mixing procedure, the initial ENM may be separated into smaller units, depending on ENM and media characteristics, energy input and research question. The latter also defines the choice of dispersion protocol, i.e. whether realistic (acceptance of larger agglomerates) or worst-case conditions (dispersion until the ‘smallest dispersible unit’ is obtained) are applied (see also figures 2 and 5). Furthermore, it is suggested to assess the stability of a NM suspension over the anticipated duration of a toxicity test (see figure 8).

Dissolution testing is only necessary in case release of ions from the NM is expected or when there are concerns regarding the toxicity of the released material.

#### 3.2.1. Representative study flow chart 1

In the present study, a CuO powder (NNV-011) has been chosen for testing the applicability of the structured approach. According to specific surface area and SEM images the powder suits the requirements of a nanomaterial as defined in S1 (suppl. figure 1): with a specific surface area of 27 m^2^ g^−1^ and a theoretical density of 6.31 g cm^−3^ a primary particle size *x*_BET_ of 35 nm is calculated (table 1). This is in good accordance with the SEM image, which shows primary particle sizes between 20 and 100 nm (figure 1). Hence, all relevant parameters according to ISO/TR13014:2013 were assessed for nCuO powder.

### 3.3. Flow chart (2) choice of test media

The choice of test media depends on the type of ENM, the research question, the choice of a test system (e.g. a cell line, an aquatic organism) and with regard to risk assessment on the use of the respective ENM. As NM and test system will interact with each other, this will affect all subsequent decisions and implies the need for tailored approaches. In principle, ENMs are added to the gas phase (e.g. air), the solid phase (e.g. feed in feeding studies, soil) or liquid phase (e.g. cell culture media, different aqueous media used in ecotoxicology). For the first two application methods, direct or indirect dosing is possible. The indirect dosing is achieved by an intermediate dispersal in liquid media before addition to either the gas or the solid phase solid phase (figure 4).

With regard to ecotoxicity testing, pre-assessment of environmental fate may guide the choice of tests by determining the partitioning behaviour of the NM, i.e. into which environmental compartment or compartments the nanomaterial will partition, and consequently which series of ecological tests are most relevant for priority testing. For example, if it is expected that a NM partitions exclusively into the water compartment, then tests with pelagic test organisms would need to be conducted. However, if strong adsorption is expected, or the nanomaterial has a very high sedimentation tendency, then it may be expected to partition into soil or sediment compartments and therefore ecotoxicity testing would be needed for those compartments [26].

In any case, the variability in the composition of different media used in testing procedures is very high, and both the dispersal procedure (e.g. use of dispersant aids) and the characterisation techniques have to be adapted on a case-by-case basis.

#### 3.3.1. Representative study flow chart 2

The subsequent toxicity tests are performed in liquid media, as toxicity to aquatic organisms shall be assessed. Two different types of media were chosen, namely ADaM, an artificial medium of known composition, and natural waters, were the most crucial parameters such as pH and DOC content were assessed. Hence, the media applied show a high variability in composition (table 2).

### 3.4. DT (3) choice of test scenario and application of NM to liquid media

The addition to the liquid phase is frequently used for *in vitro* studies and aquatic ecotoxicological tests. An ENM powder may either be directly added to the respective liquid, or a predispersion (stock solution) in water is prepared. As specified in figures 2 and 5, toxicity testing of ENMs can be conducted under different preconditions: (1) avoidance of NM agglomeration (‘worst-case’) or (2) acceptance of agglomeration (‘realistic conditions’). These two scenarios result in different preliminary demands and considerations regarding toxicity testing. Depending on the scenario chosen, the use of dispersing agents in order to keep particles separated from each other is an option. The characteristics of a given NM may differ due to the medium composition, with factors like pH, salt content, use of dispersant aids, presence of chelators influencing NM behaviour, making it inevitable to assess the characteristics of individual NM-medium combinations. In order to demonstrate the implications in detail, both scenarios are covered by the CuO representative study.

In some cases, the dispersion of a nanomaterial in liquid media is impossible, e.g. for powders with a poor wettability. Without dispersant aids no further testing in liquid media is possible, however this does not affect NM powders to be tested in aerosols and soils and sediments (figure 5).

#### 3.4.1. Representative study DT 3

The logical sequence of decisions taken within DT 3 for the example of nCuO is depicted in supplement figure 3.

The nanoscale CuO was tested under relevant conditions, by using it in its non-stabilized form in the Daphnia OECD standard test, and in a test using natural waters. A worst-case scenario is represented by stabilizing the CuO particles in ADaM with 0.1 wt% of the dispersant tetrasodium pyrophosphate (TSPP; CAS 13472-36-1). The stabilisation has a tremendous impact on the long-term suspension stability; achieving constant exposure conditions over 72 h, whereas without the TSPP, the particles start to agglomerate immediately upon addition to the media.

For the CuO nanoparticles an indirect dosing procedure was chosen and a pre-dispersion in water was prepared, which was dosed to the respective test medium. The advantage of this approach is the application of an identical dispersion procedure for all media. In order to cover both, the realistic and the worst-case scenario, the pre-dispersion (stock suspension) was prepared with and without the dispersing agent TSPP. While the impact of stabilisation on *x*_DLS_ was not significant (see table 1), the absolute value of the zeta potential rose from around +30 mV for the unstabilized nCuO to around −65 mV for the stabilized stock suspension, implying a substantial increase in stability (figure 1). The use of stabiliser also had an impact on long-term stability of the stock suspension, as in the presence of TSPP, repeated preparation of identical nCuO suspensions in ADaM was achieved up to a stock suspension age of 96 h, leading to an increase in reproducibility (data not shown). Neither without TSPP, nor by mixing nCuO, ADaM and TSPP upon start of the toxicity test, a stable nCuO suspension was achieved. When dispersing unstabilized stock suspension in ADaM or lake water, nCuO starts to agglomerate immediately, making the determination of the zeta potential impossible (table 3).

**Table 3.** Characteristics of nanoscaled CuO in the ADaM test media under realistic and worst-case conditions.

Properties/characteristics	CuO NNV-011	CuO NNV-011 + 0.1% TSPP
Particle size in ADaM after dispersion (at *t* = 0; smallest dispersable unit) *x*_DLS_	Not measurable	132 ± 5 nm
Zeta potential in ADaM	Not measurable	−64 mV

### 3.5. DT (4) addition of ENM to the gas phase

The addition of ENMs to the gas phase, e.g. for inhalation studies requires the generation of an aerosol containing the ENM. This can be achieved by either using an ENM powder (direct dosing) or an ENM suspension (indirect dosing). In any case, the respective dispersion procedure needs to be specifically adapted to the shape of the nanomaterial under study, as particles, fibres and plates will disperse and behave differently in the gas phase (figure 6). In addition, changes in NM properties upon contact with biological fluids, such as lung surfactant, need to be taken into account e.g. [27]. In the example of nanoscale CuO employed in aquatic toxicity tests, this tree is not relevant.

### 3.6. DT (5) addition of ENM to the solid phase

If tests are conducted in soils and sediment, basic data on NM physicochemical properties is often the sole information on ENM characteristics because there is a lack of techniques to characterize NM in these compartments.

ENM addition to the solid phase is required e.g. in feeding studies or for soil and sediment testing. Direct (dry-spiking, powder) and indirect (wet-spiking, suspension) methods can be used. The latter method requires the preparation of a NM suspension from a powder, which is then added to the solid (figure 7). In this case, it is possible to assess NM characteristics in liquid before mixing with the solid matrix. The aging/pre-treatment step after the addition of NM is suggested according to McShane *et al* [28].

[Fig F0008]The analytical techniques to determine ENM characteristics in solid test media are still inadequate, resulting in a lack of information on behaviour and interactions of ENMs and the solid matrix (e.g. Kühnel and Nickel [12]). This is marked as ‘area of research’ in figure 7, as future method development may allow a specification of the procedure. In the example of nanoscale CuO employed in aquatic toxicity tests, this tree is not relevant.

### 3.7. DT (6) dose metrics in liquid media

The determination of ENM dose in liquid media is important to interpret toxicological results and on a regulatory basis has a high significance to determine toxic concentrations and e.g. exposure limits. The suitability of mass-based dose metrics is a scientific matter of debate and number- or surface-base dose metrics have been suggested [12, 29]. In any case, knowledge on initial ENM properties and characteristics need to be as extensive as possible. In addition ENM behaviour in a given test, specifically parameters such as agglomeration, dissolution, sedimentation, have to be taken into account.

#### 3.7.1. Representative study DT 6

The nCuO suspensions were prepared according to mass concentration, and depending on exposure scenario chosen and the type of test medium used, the particles underwent agglomeration resulting in sedimentation of particles (table 4).

**Table 4.** Agglomeration and dissolution behaviour of nCuO in ADaM with 0.1% TSPP over 48 h. After 48 h of incubation, strong agglomeration and sedimentation of nCuO particles occurred, making the determination of zeta potential impossible.

*t* (h)	*X*_DLS_ (nm)	Zeta potential (mV)	Dissolved Cu (mg l^−1^)
0	132	−64.3	3.5
6	132	−41.3	4.63
24	146	−38.3	5.41
48	230	Not measurable	6.1

*Daphnia magna* is a free-swimming filter feeder and hence contacts nCuO suspended in the water phase as well as larger particles deposited on the bottom of the test vessels. Dissolution plays a role for CuO particles, but was low for the nanomaterial under study (table 4). Hence, for the effect values determined in the different toxicity tests, mass-based dose metrics were chosen. The effect values (EC_50_) for the different media and test scenarios are presented in table 5, in the case of nCuO the unstabilized form (representing the ‘realistic’ scenario) was the most toxic, whereas the test under stabilized conditions (representing the ‘worst-case’ scenario) showed the lowest toxicity.

**Table 5.** Half maximal effective concentrations EC_50_ (mg l^−1^) in *Daphnia magna* after 48 h exposure to nanoscale CuO in different media.

CuO + 0.1% TSPP (in ADaM)	CuO (unstabilized) (in ADaM)	Lake water I	Lake water II
11.23	0.44	5.64	6.84

## 4. Discussion

When choosing a test design for assessing NMs hazard towards organisms, various considerations on all steps of the testing procedure have to be taken into account, namely before (e.g. preparation of suspensions), during (behaviour of ENMs in the test media over time) and after conducting a test (NM re-characterisation, data interpretation). Here, the suitability of DT and flow charts as a tool to support a consistent and structured testing of ENM was explored. They provide a stepwise approach, dividing a method or process into crucial steps. Further, they allow identifying the most favourable option for a specific research question out of two or more alternatives. The structured approach is intended to be used for testing with both scientific as well as regulatory background.

The need for test procedures specific to ENMs arises from their unique properties and behaviour, which in many aspects differs substantially from that of conventional chemicals. The experience from numerous studies has shown that the translation of test protocols developed for chemicals is not recommendable. Rather, the test design for ENMs has to be reconsidered and amendments need to be implemented [12, 30]. Additionally, the large variety of NM tested implies that testing schemes need adoptations considering the specific peculiarities of a given nanomaterial. For example, the preparation of suspensions requires material specific procedures, mostly involving energy input of varying intensity and therefore different side effects like radical formation or wear debris from the probe tip can occur [31, 32]. In consequence, a structured approach to take controlled decisions on all necessary steps, such as characterisation procedures, dispersal and the actual toxicity testing to assess the hazard of a NM, is proposed here involving a series of DT and a flow chart.

In order to prove the applicability and correctness of the logical sequence in which decisions are taken, an example study employing a CuO powder was conducted, and different preconditions for testing were assumed. From the different testing scenarios, the need to disperse the CuO NM in test media showing a high variance in compositions arose, either with or without stabilisers. The DT and flow charts were developed to cover these broad demands and were found to perform well. For the test scenario avoiding the use of stabilisers, agglomeration and sedimentation processes hinder the proper assessment of NM behaviour in media, as with the techniques applied neither the zeta potential nor particle size were measurable (table 3). As demonstrated in table 5, the different test conditions had a clear influence on the effect values observed in the toxicity tests, with EC_50_ values for nCuO ranging from 0.44 to 11.23 mg l^−1^. Hence, the DT were found to be helpful in defining a test scenario, in developing a test protocol (SOP) as well as in interpreting final test results due to detailed knowledge on NM characteristics under the respective test condition. For SOP development, following the logical sequence specified by the DT prevents inconsistencies and errors in the experimental design. Likewise, the DT and flow charts may be used to assess the reliability of existing data on NM. In that sense, the structured approach is also supporting the specification of established test guidelines with regard to the testing of nanomaterials, fostering harmonisation of NM testing in the future [33]. Beyond the example of CuO, DT help in making material specific decisions whenever needed e.g. when aging processes become relevant. Hence, the DT and flow charts are applicable to various NM with different prerequisites regarding composition and state (powder/suspension).

In case of information gaps, passing through a tree may become impossible and hence the information has to be retrieved before conducting any test. This also fosters the assessment of minimal NM properties and characteristics before actually testing an NM, and a growing database on NM physicochemical properties will allow to better link NM properties to observed toxicological effects. With regard to intelligent testing strategies for nanomaterials, the linkage between NM properties and toxicological outcome is valuable for the development of grouping strategies for nanomaterials [3, 34].

## 5. Conclusions and outlook

A proposal for defined procedures in toxicity testing of NM was presented by structuring all relevant considerations in the frame of DT and flow charts. They were found to be a versatile tool applicable to different testing preconditions and scenarios. Likewise, DT are adaptable and flexible, hence being suitable for different types of NM and allowing the integration of new knowledge.

The structured approach presented here provides a basic framework and both the degree of detail and the extent can be further elaborated, or integrated into additional DT. Future issues to be integrated into the DT include those marked as ‘areas of research’ in the proposed DT, for example aging and sedimentation processes, and issues not yet considered such as transformation and degradation processes (e.g. coating), and the selection of proper controls for toxicity testing.
